# Wastewater Membrane Bioreactors: A Comprehensive Review of Explainable Artificial Intelligence and Digital Twin Applications

**DOI:** 10.3390/membranes16050181

**Published:** 2026-05-21

**Authors:** Wael S. Al-Rashed

**Affiliations:** Department of Civil Engineering, Faculty of Engineering, University of Tabuk, P.O. Box 741, Tabuk 71491, Saudi Arabia; walrashed@ut.edu.sa

**Keywords:** membrane bioreactor, explainable artificial intelligence, digital twin, membrane fouling, SHAP, energy optimization, machine learning, wastewater treatment

## Abstract

Wastewater membrane bioreactors (MBRs) have become an important advanced treatment technology due to their ability to produce high-quality effluent suitable for discharge and water reuse. However, their broader and more sustainable application remains constrained by membrane fouling, elevated energy demand, and the operational complexity of coupled biological and membrane separation processes. This comprehensive review critically evaluates the growing application of machine learning (ML), explainable artificial intelligence (XAI), and digital twin (DT) technologies in MBR systems. Published studies on fouling prediction, energy optimization, effluent quality estimation, and intelligent operational support are critically evaluated, with explicit attention to model performance, dataset limitations, and generalizability. The reviewed literature shows that ML models, particularly ensemble methods, support vector machines, and deep learning approaches, have demonstrated strong potential for predicting major MBR performance indicators, including transmembrane pressure, permeate flux, fouling resistance, and selected effluent-quality variables. In parallel, XAI methods such as SHAP, LIME, and Anchors are increasingly being used to enhance model transparency and to reveal the dominant factors controlling process performance. Digital twin frameworks further extend this potential by enabling the integration of mechanistic understanding, online sensor data, data-driven prediction, and interpretable decision support within real-time operational platforms. Nevertheless, several barriers continue to hinder practical implementation, including the limited number of full-scale studies, the scarcity of openly accessible and standardized datasets, insufficient consideration of uncertainty and model drift, and the early-stage maturity of DT deployment in operational plants. The evidence reviewed suggests that integrating ML, XAI, and DT can substantially improve the reliability, interpretability, and operational efficiency of MBR systems. Future research should therefore focus on full-scale validation, the development of benchmark datasets, uncertainty-aware modeling, and practical deployment strategies for interpretable intelligent MBR management.

## 1. Introduction

Global water scarcity and increasingly stringent effluent discharge regulations have elevated membrane bioreactor (MBR) technology from a niche research application to a mainstream wastewater treatment solution deployed on every inhabited continent [1,2]. MBRs couple activated sludge biological treatment with pressure-driven membrane filtration. Hollow-fiber or flat-sheet ultrafiltration membranes are submerged directly in the bioreactor. MBRs produce a consistently high-quality, pathogen-free, and nutrient-reduced permeate. This effluent meets standards for direct non-potable reuse in agricultural irrigation, industrial process water, and environmental discharge [3,4]. This combination of compact footprint, flexible operation, and superior effluent quality has driven widespread commercial adoption. MBR systems now operate at scales ranging from decentralized 10 m^3^/day units to large municipal installations treating over 100,000 m^3^/day [1,5].

Since the first commercially significant installations in the early 1990s, MBR technology has expanded rapidly. More than 5000 wastewater treatment plants worldwide now use MBRs across municipal, industrial, and water-reuse applications [6,7]. Industrial applications span food and beverage processing, pharmaceutical manufacturing, textile and dye production, and petrochemical wastewater treatment. Each sector presents distinct mixed-liquor compositions and fouling challenges that conventional treatment technologies cannot address within the same process footprint [3,4]. The regulatory landscape has also shifted in MBR’s favor. Effluent standards for nitrogen, phosphorus, and emerging contaminants have become increasingly stringent. Requirements for treated water reuse have also expanded. In many markets, MBR technology is now the only technically feasible option [2].

Despite this commercial success, MBR technology carries two persistent structural liabilities that constrain further deployment. Membrane fouling is the progressive accumulation of organic foulants, colloidal particles, and microbial extracellular polymeric substances (EPS) onto membrane surfaces. Within pore structures, fouling also reduces permeate flux, elevates transmembrane pressure (TMP), triggers chemical cleaning events, and ultimately forces premature membrane replacement. This represents the dominant lifecycle cost driver for MBR installations [4,8,9]. The fundamental mechanisms governing fouling are complex, nonlinear, and highly sensitive to mixed-liquor composition, sludge age, operational flux history, and hydraulic conditions. Even small changes in influent characteristics can significantly shift fouling behavior within a single operating day [10,11,12]. The second liability is energy. Aeration for biological oxygen transfer and membrane scouring, combined with permeate pumping, collectively accounts for 0.4–1.5 kWh/m^3^ of treated water. This is roughly double the specific energy demand of conventional activated sludge processes. Membrane aeration alone represents 60–75% of that total [13,14,15]. At scale, this energy penalty translates to substantial operating costs and carbon footprints that undermine the environmental credentials of water reuse applications.

Fouling unpredictability and energy inefficiency are precisely where machine learning offers a decisive advantage. ML algorithms learn complex, non-linear relationships between operational variables and process outcomes directly from historical data. They do not require the full mechanistic parameterization that constrains physics-based models in operational settings [16,17]. Support vector machines [18], random forests, gradient-boosting frameworks [19], and deep recurrent networks [20,21] have been applied to MBR systems. These approaches have demonstrated substantially better predictive accuracy than linear regression and simplified mechanistic models, across diverse scales and wastewater types [22,23,24,25,26].

However, deploying ML in regulated water treatment environments faces a critical barrier: the opacity of black-box model predictions. Operators, process engineers, and regulators require evidence that model recommendations are physically meaningful and consistent with domain knowledge. They also need assurance that models will not produce catastrophically incorrect outputs under unusual conditions. XAI tools such as LIME and gradient-based attribution methods address this need [27,28]. These tools decompose model predictions into quantifiable feature contributions. Engineers can interrogate model behavior, validate predictions against domain knowledge, and build operator trust. They also help satisfy the regulatory expectation for explainable automated decisions in critical infrastructure [29].

A third technological trajectory has emerged: the digital twin. Digital twins serve as an integration framework that combines ML prediction, XAI transparency, and mechanistic modelling within a real-time operational tool [30,31,32]. A digital twin is a continuously updated, bidirectionally coupled virtual replica of a physical system. It combines high-fidelity process models with real-time sensor data and data-driven corrections. This enables predictive simulation, scenario testing, and optimization without physical experimentation [33]. For MBR systems, a mature digital twin would integrate activated sludge models [34,35], membrane filtration sub-models [36], ML-based fouling predictors, XAI explanation modules, and energy optimization engines. Together, these form a unified platform that provides operators with both operational recommendations and the transparent reasoning behind them [37].

No prior review has thoroughly mapped the intersection of ML, XAI, and DT as an integrated analytical framework for MBR systems. Existing reviews address ML and data-driven approaches in MBR and membrane processes in isolation [24,38], or examine DT applications in wastewater treatment without incorporating XAI transparency [37]. The present work is the first to synthesize all three paradigms within a single critically evaluated framework, directly addressing the gap between predictive capability and interpretable, deployable intelligent MBR control. The three paradigms form a logical and sequential chain. ML provides the predictive engine: it learns complex non-linear relationships from operational data and forecasts fouling, energy demand, and effluent quality with an accuracy that deterministic models cannot match under dynamic real-world conditions. XAI provides the interpretability layer: it decomposes individual ML predictions into ranked feature contributions, making model outputs auditable by engineers and regulators. Without XAI, ML cannot be safely deployed in regulated infrastructure. DT provides the operational integration layer: it combines ML predictions, XAI explanations, and mechanistic process models within a continuously updated virtual replica of the physical plant, enabling predictive simulation and scenario testing without physical experimentation. Each layer depends on the one before it. A predictive ML model without an XAI layer cannot satisfy regulatory transparency requirements. An XAI layer without a DT framework provides only post-hoc explanations without closed-loop operational integration. Only the combination of all three creates a system capable of trustworthy, interpretable, and deployable autonomous MBR control. This interdependence is the central analytical argument of this review and defines the scope of the critical synthesis that follows.

**Figure 1:**
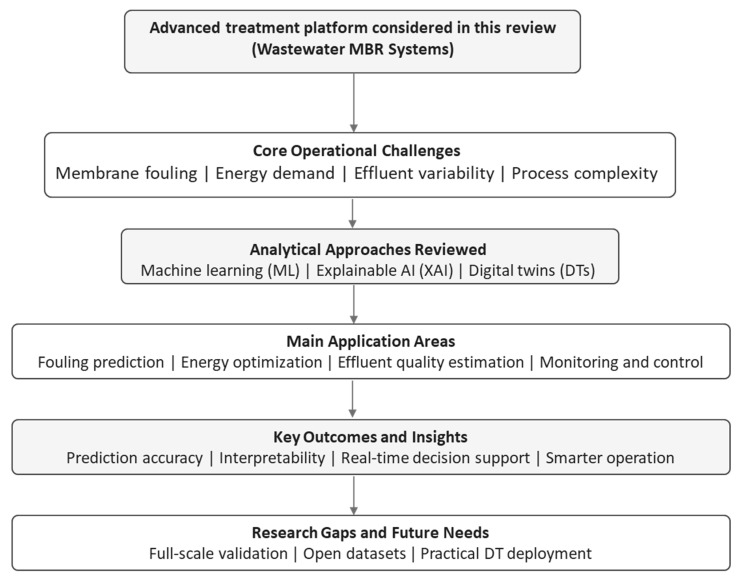
Conceptual framework of the review, showing the main operational challenges in wastewater membrane bioreactors (MBRs), the analytical paradigms covered in this paper (machine learning, explainable artificial intelligence, and digital twins), their major application areas, and the key research gaps and future directions identified.

This review addresses these gaps through four objectives. First, it critically evaluates ML model performance, dataset limitations, and generalizability across MBR fouling prediction, energy optimization, and effluent quality estimation. Second, it assesses XAI interpretation frameworks and their practical role in building trustworthy, operator-accessible decisions for MBR systems. Third, it characterizes emerging digital twin architectures, their deployment maturity, and the requirements for integrating XAI within operational DT platforms. Fourth, it identifies the critical research gaps that must be resolved before XAI-DT integration can achieve operational-scale deployment. Figure 1 presents a schematic overview of the main MBR operational challenges, analytical paradigms, application domains, and key research gaps identified in this review.

Following this framework, the subsequent sections describe the review methodology and then critically discuss ML, XAI, and DT applications in wastewater MBR systems.

It should be noted that the term ‘comprehensive’ in the title refers to the breadth of the paradigms reviewed, spanning ML, XAI, and DT within a single analytical framework, rather than implying that the field itself is mature at an operational scale. Indeed, the scarcity of full-scale DT deployments and the limited extent of XAI integration documented throughout this review are among the review’s central findings and define the research agenda articulated in Section 7.

## 2. Methodology

### Literature Search and Study Selection

A focused literature search was conducted across three major scientific databases: Scopus, Web of Science, and PubMed. The search covered the period January 2010 to December 2025. This lower bound was chosen because it coincides with the period of significant growth in both MBR commercial deployment and the application of ML to environmental engineering.

The search used free-text keywords combined with Boolean operators: (“membrane bioreactor” OR “MBR”) AND (“machine learning” OR “artificial neural network” OR “deep learning” OR “LSTM” OR “random forest” OR “support vector machine” OR “explainable AI” OR “XAI” OR “SHAP” OR “LIME” OR “digital twin”). The search was restricted to peer-reviewed journal articles in English. Reference lists of retrieved articles were checked manually for additional relevant sources. Studies were excluded if they lacked quantitative performance metrics or focused solely on membrane material fabrication.

The studies retained after this search form the evidence base of this review. This work is a narrative review focused on AI trends in MBR systems. It does not constitute a systematic review or meta-analysis, and accordingly a PRISMA flow diagram was not established. Studies were retained if they addressed ML, XAI, or DT applications in MBR systems and reported original modelling or empirical results. Study-level data for all reviewed primary studies, including target variable, unit, reported value range, performance metric, and external validation approach, is provided in Supplementary Table S1.

## 3. Machine Learning for Membrane Fouling Prediction

### 3.1. Fouling Mechanisms and Modelling Context

Membrane fouling in MBRs is a multi-scale, multi-mechanism phenomenon. Its overall resistance is partitioned using the resistance-in-series framework into three contributions: reversible cake layer formation (removable by relaxation or backwashing), irreversible pore blocking (requiring chemical cleaning), and adsorptive fouling within the membrane matrix [4]. The contribution of each mechanism depends on mixed-liquor composition and on the operating flux relative to the critical flux threshold. Key EPS components include soluble microbial products (SMP), bound EPS, and colloidal biopolymers [39]. Below the critical flux, fouling accumulation is gradual and largely reversible; above it, rapid and irreversible fouling occurs, dramatically shortening cleaning intervals and membrane lifetime [4,9].

Several operational parameters govern fouling kinetics. MLSS concentration determines the mass of material available for cake formation and influences mixed-liquor viscosity. SRT controls EPS production through its effect on biomass growth rate and substrate availability. HRT affects the dilution and residence time of colloidal foulant material. DO concentration governs the balance between aerobic and anoxic metabolic pathways, which shapes EPS composition. Membrane aeration intensity provides the shear stress required to limit cake layer growth on submerged membrane surfaces [11,12]. The interactions between these variables are non-linear and frequently antagonistic. For example, increasing MLSS improves biological treatment performance. However, it accelerates fouling, while increasing aeration reduces fouling but increases energy consumption, creating multi-objective trade-offs that are difficult to capture in simple mechanistic models [23].

These operational parameters, namely MLSS, SRT, HRT, DO, and aeration intensity, also constitute the primary candidate feature set for ML models targeting MBR fouling prediction. Their selection as model inputs is therefore grounded in mechanistic understanding rather than empirical convention. This linkage between process knowledge and feature engineering is a key strength of the hybrid modelling approaches discussed in Section 3.2.

Mechanistic models, including the ASM family [34,35] and the BSM-MBR benchmark [36], have substantially advanced understanding of biological kinetics and their coupling to membrane performance. However, their predictive accuracy under dynamic loading conditions is limited by several factors. These include the difficulty of characterizing mixed-liquor EPS fractions in real time, the strong sensitivity of model parameters to temperature and sludge history, and the high computational cost of full mechanistic simulation at operational timescales [23,40]. Osmotic MBR (OMBR) configurations introduce additional complexity by coupling the bioreactor to the forward osmosis (FO) membrane via an osmotic driving force, creating concentration polarization dynamics that further motivate data-driven approaches [22,41]. These limitations have motivated the ML approaches reviewed in the following subsections.

**Figure 2:**
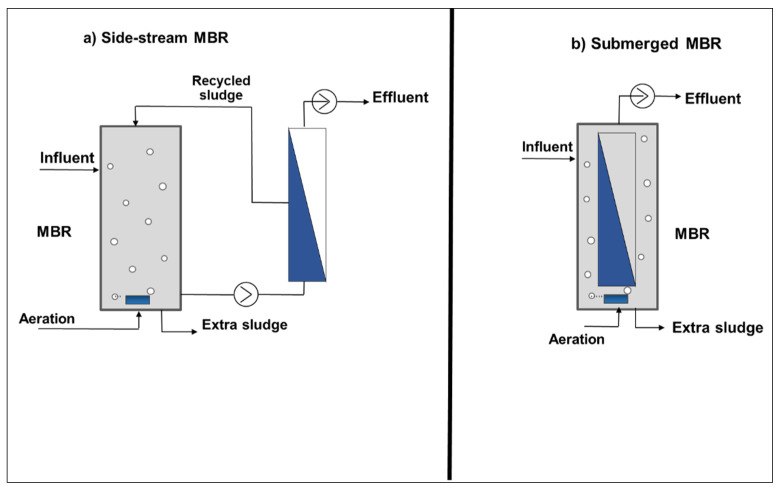
Schematic of a typical submerged and side-stream wastewater membrane bioreactor (MBR) system.

To provide a concrete operational reference for the parameters and data streams discussed in the following sections, Figure 2 presents a schematic of a typical submerged and side-stream MBR system. The diagram shows the key unit operations. These include the aerated bioreactor with activated sludge mixed liquor, the submerged hollow-fiber or flat-sheet ultrafiltration membrane module operating under negative permeate pressure, the permeate extraction pump and effluent outlet, the sludge recycle line maintaining the target MLSS concentration, and the waste sludge outlet controlling SRT. The coarse-bubble membrane aeration system provides shear stress for fouling control and for the backwash and chemical cleaning (CIP) circuits. The principal online sensor measurement points are also indicated: dissolved oxygen probes in the bioreactor, transmembrane pressure transducers on the permeate line, influent and permeate flow meters, online turbidity sensors, and temperature probes. The data pathway from these field sensors through the SCADA data acquisition system to the ML prediction and digital twin analytical layers is shown, establishing the physical and data context for the modelling work reviewed in Section 3, Section 4, Section 5 and Section 6.

### 3.2. Shallow and Kernel-Based ML Models

The studies reviewed in this section were selected because they met all the inclusion criteria defined in Section Literature Search and Study Selection, including reporting at least one quantitative performance metric and applying original ML methodology to MBR operational or experimental data. Direct benchmarking across studies is constrained by heterogeneity in target variables, dataset sizes, and operating conditions; performance metrics should therefore be interpreted within each study rather than as absolute rankings.

Artificial neural networks were among the first ML architectures applied systematically to MBR fouling prediction. Mirbagheri et al. compared multi-layer perceptron (MLP) and radial basis function (RBF) neural networks for TMP and permeability prediction in a pilot-scale submerged MBR, using time, TSS, COD, SRT, and MLSS as input variables across a 60-day operating campaign [23]. Both architectures produced satisfactory predictions, with RBF networks demonstrating faster convergence and reduced sensitivity to initial weight conditions, an important practical consideration for online model deployment. The study highlighted the importance of selecting input variables and ensuring diversity in the training dataset for generalization beyond the training period. Schmitt et al. subsequently developed a backpropagation ANN for fouling prediction in an anoxic-aerobic MBR treating domestic wastewater. The model achieved R^2^ = 0.850 on held-out test data, reflecting the inherent variability of pilot-scale operation and the challenge of capturing fouling dynamics with limited input variables [25].

Kernel-based methods, which map input data to higher-dimensional feature spaces to learn non-linear decision boundaries without deep architectures, have demonstrated competitive performance. Hamedi et al. conducted a rigorous benchmarking study comparing ANN-MLP, ANN with particle swarm optimization (ANN-PSO), gene expression programming (GEP), and least-squares support vector machine (LSSVM) for fouling resistance prediction in a laboratory MBR [42]. LSSVM achieved the best performance with R^2^ = 0.990 and MSE = 0.0002, substantially outperforming all ANN variants. Sensitivity analysis identified permeate flux and TMP as the dominant input variables, consistent with the resistance-in-series framework, demonstrating that ML feature importance analysis can recover mechanistically meaningful variable rankings. Giwa et al. applied ANN modelling to a submerged MBR treating mixed industrial and municipal wastewater in the UAE. They showed that feed water characteristics (conductivity, pH, suspended solids) are important predictors of effluent quality parameters, including COD, BOD, and turbidity. ANN performance depended critically on training data diversity across different loading conditions [43]. More recent work has confirmed and extended these findings. Nguyen et al. applied decision tree regression, support vector regression, and linear regression to predict TMP in a domestic wastewater MBR [44]. Decision tree regression achieved R^2^ = 0.99. This result must be interpreted with caution. The dataset was small and single-facility. The high R^2^ likely reflects fitting to that dataset’s specific noise structure rather than a generalizable fouling model. A comprehensive review by Queiroz et al. analysed 57 ML studies on MBR performance prediction [45]. ANNs were used in 88% of cases. The review also identified a critical gap: no study had used ML to predict membrane lifespan or replacement timing. This gap has direct economic relevance for plant operators.

Survey studies have systematically evaluated the state of ML modelling in MBR research. Schmitt and Do reviewed ML approaches for MBR fouling modelling across 30+ studies, identified data availability and representativeness as the primary constraints on model generalizability, and recommended long-duration continuous monitoring campaigns as the minimum dataset requirement for reliable model development [24]. Shi et al. reviewed ML applications across membrane filtration processes more broadly and concluded that hybrid ML–mechanistic models, which use mechanistic equations to compute derived input features before ML prediction, consistently outperformed purely black-box approaches in cross-validation studies, particularly when extrapolating beyond the training operating range [38].

### 3.3. Ensemble Methods and Deep Learning

Ensemble methods, algorithms that combine predictions from multiple base learners to reduce variance and improve generalization, have produced the strongest overall performance in the MBR ML literature. The random forest (RF) algorithm [21] aggregates predictions from an ensemble of independently trained decision trees, each built on a random subset of training data and input features. RF is particularly well-suited to MBR fouling prediction because it handles mixed variable types, is robust to outliers in process data, and provides built-in feature importance estimates without requiring a separate XAI step. Random forests are not without limitations, however. Their memory footprint scales with ensemble size, which can restrict deployment on embedded or edge hardware in real-time MBR control systems. Feature importance rankings from RF are also global and may mask local non-linearities; SHAP-based attribution, discussed in Section 4, is therefore recommended alongside RF when interpretability is required.

Viet and Jang demonstrated the potential of AI-based models for predicting osmotic MBR performance, applying several architectures to an OMBR system treating municipal wastewater [22]. Using feed water quality parameters, pH, conductivity, NH_4_-N, total nitrogen, and total organic carbon, as model inputs, their best-performing models achieved R^2^ = 0.92–0.98 for water flux and fouling resistance prediction, capturing the complex osmotic driving force dynamics that govern OMBR performance. The results demonstrated that data-driven approaches can navigate the OMBR system’s additional degrees of freedom, account for solution concentration and dilution effects, and capture reverse salt flux, without requiring explicit mechanistic parameterization of FO transport equations.

Kovacs et al. conducted the most comprehensive full-scale ML validation yet published in the MBR domain, applying RF, ANN, and LSTM models to a dataset comprising more than 80,000 samples from a municipal wastewater treatment plant [26]. The RF model achieved R^2^ = 0.927–0.996 and RMSE = 0.264–0.904 kPa across the different stages of the MBR filtration cycle (initial fouling, stable operation, late-stage compaction, and post-cleaning recovery). ANN and LSTM produced higher overall RMSE values than RF. However, the LSTM showed particular strength in the temporally structured late-stage fouling period, where the history of flux and TMP evolution carries significant predictive information for the current fouling trajectory. Importantly, this study was the first to validate ML TMP prediction at full municipal scale with this level of accuracy, providing one of the strongest full-scale demonstrations to date of ML-based TMP prediction. It is critical to note, however, that validation on historical SCADA data is not equivalent to closed-loop operational deployment. Historical validation confirms that a model can reproduce past patterns from a single facility. It does not confirm that the model will perform reliably under real conditions involving sensor drift, measurement noise, data latency, or equipment failures. Full operational deployment requires additional closed-loop testing under live conditions before model outputs can be relied upon for operational decision-making.

It should be noted, however, that this dataset originates from a single municipal plant operating under a specific membrane configuration, sludge regime, and local influent composition. Operational variability arising from seasonal temperature fluctuations, industrial discharge events, and membrane aging was not separately analyzed. The generalizability of these models to other MBR facilities, therefore, remains to be established through cross-site validation, as discussed in Section 3.4.

The practical feasibility of LSTM and Transformer architectures in operational MBR systems warrants careful consideration. LSTM networks require substantially larger training datasets than shallow models, typically thousands to tens of thousands of time steps, to learn meaningful temporal dependencies without overfitting. Their training and inference also impose greater computational demands than random forests or support vector machines, which may limit deployment on the embedded industrial controllers typically used in MBR plants. Transformer architectures present an even higher data and compute burden. Model compression techniques, including pruning, quantization, and knowledge distillation, offer pathways to lightweight deployment but have not yet been evaluated in the MBR context. These constraints should be explicitly reported in future deep learning studies to allow practitioners to assess deployment readiness.

The emergence of LSTM networks [20] is particularly significant for MBR fouling modelling because TMP evolution is inherently time-dependent: the current fouling state encodes the accumulated history of flux excursions, aeration cycles, and sludge condition over preceding hours and days. LSTM networks, with their gated memory cells that selectively retain long-range temporal dependencies, are well-suited to this problem. Gradient boosting frameworks, such as XGBoost and LightGBM, have achieved strong performance on broader water-quality prediction tasks, and extending them to MBR-specific fouling prediction with appropriate temporal feature engineering represents a natural next step. Transformer architectures [46], which use self-attention mechanisms to capture global dependencies across input sequences, have demonstrated state-of-the-art performance in environmental time-series forecasting and represent a frontier application for MBR fouling prediction that has not yet been systematically evaluated in peer-reviewed studies. Random forests are not without limitations, however. Their memory footprint scales with ensemble size, which can restrict deployment on embedded or edge hardware in real-time MBR control systems. Feature importance rankings from RF are also global and may mask local non-linearities; SHAP-based attribution, discussed in Section 4, is therefore recommended alongside RF when interpretability is required.

### 3.4. Dataset Limitations, Overfitting Risk, and Cross-Site Generalization

A critical appraisal of the performance metrics summarized in Table 1 must account for the substantial heterogeneity in dataset characteristics across reviewed studies. The majority of studies trained and validated models on single-facility datasets comprising hundreds to a few thousand samples. Only Kovacs et al. reported a dataset comprising more than 80,000 operational samples from a full municipal-scale plant. High reported R^2^ values, including R^2^ = 0.990 for LSSVM [42] and R^2^ = 0.92–0.98 for OMBR prediction models [22], must therefore be interpreted with care. When a model is trained and tested on data from a single, homogeneous operating campaign, a high R^2^ can reflect the model’s capacity to reproduce the specific noise structure and temporal autocorrelation of that dataset rather than a genuinely generalizable representation of fouling dynamics. This overfitting risk is compounded by the near-universal absence of external validation on data from a separate operating period or independent plant; most reviewed studies relied on random train–test splits that do not preserve temporal ordering and therefore cannot detect temporal data leakage. Future studies should report k-fold cross-validation with temporal blocking, learning curves as a function of training set size, and, where feasible, cross-site validation on at least one independent facility.

The ability of a model trained at one MBR installation to maintain predictive accuracy when applied to a second facility with different membrane module geometry, influent composition, or operating regime, referred to here as cross-site generalization, has rarely been evaluated in the reviewed literature. Dataset shift, arising from differences in sludge microbiology, local wastewater chemistry, and climate-driven seasonal patterns, is the primary mechanism driving cross-site performance degradation. Transfer learning, in which a model pre-trained on a data-rich source facility is fine-tuned with limited data from a target plant, and domain adaptation, which explicitly minimizes the distributional discrepancy between source and target feature spaces, offer technically feasible pathways toward generalizable MBR ML models. Both approaches have demonstrated success in related environmental engineering contexts [17,47] and represent a high-priority methodological direction for future MBR ML research.

The limitations and generalizability constraints identified in this section directly affect the adoption of ML in regulated MBR operations. Practitioners and regulators require not only accurate predictions but also transparent, auditable justification for model-driven recommendations. This requirement motivates the use of explainable AI methods, which are evaluated in the following section. Table 2 presents a critical comparison of the principal ML algorithms reviewed, based solely on verified data from the primary cited sources. The table highlights the key strength, the primary limitation, and the bias risk level of each algorithm. Bias risk is rated High where no external validation on an independent facility was reported and the dataset comprised fewer than 500 samples. It is rated Moderate where the dataset was large but validation remained within a single facility. It is rated Low where at least two independent test sets from operational plants were used. This assessment follows the framework proposed by Reviewer and by published guidance on ML validation standards in environmental engineering [28,48].

## 4. Explainable Artificial Intelligence in MBR Applications

### 4.1. The Explainability Imperative in Regulated Water Systems

The adoption of ML in regulated water treatment environments faces a challenge that does not arise in most commercial ML applications: operators and regulators require not only accurate predictions but also interpretable justification for model-driven recommendations. In a manufacturing setting, a black-box ML system that reduces costs can be adopted solely on performance grounds. In a water utility, the bar is higher. An ML system that recommends reducing membrane aeration must demonstrate that the recommendation is physically sound, that it will not cause irreversible fouling under current conditions, and that it will not result in permit violations. All of this must be verifiable within the timeframe of the operating shift [29,51]. In regulated water treatment environments, the practical adoption of ML recommendations depends not only on predictive performance but also on operator trust, engineering plausibility, and the ability to document decision rationale.

Rudin [51] argues, in a widely cited analysis, that in high-stakes sequential decision-making domains, inherently interpretable models should be preferred over post-hoc explanations of black-box models whenever the mapping is sufficiently complex. However, the non-linear, multi-variable, temporally dependent nature of MBR fouling dynamics generally exceeds the representational capacity of interpretable models (e.g., logistic regression, decision trees) to achieve adequate predictive accuracy for operational use, making post-hoc XAI explanations of high-performing black-box models the pragmatic choice for the field [48]. XAI methods bridge this gap by providing explanations in a form that domain experts can evaluate, validate against process knowledge, and act upon, enabling the combination of black-box predictive power with the interpretability required for regulated deployment [29].

### 4.2. SHAP: Dominant XAI Framework in MBR Studies

SHAP (SHapley Additive exPlanations) [52], grounded in cooperative game theory, has emerged as the dominant XAI framework applied to MBR studies. Across XAI-inclusive publications identified in this review, SHAP was the most frequently applied method by a considerable margin. SHAP assigns each feature a contribution value equal to its expected marginal contribution across all possible feature subsets. This provides three desirable properties for engineering applications: local accuracy (SHAP values sum to the model output), consistency (features with larger marginal effects receive larger SHAP values), and missingness (absent features contribute zero). These properties make SHAP values directly interpretable as feature-level contributions to each prediction, enabling both local (per-prediction) and global (dataset-wide) model explanations [53].

Across the limited MBR studies that have applied SHAP or related feature-attribution methods, operational variables such as MLSS, SRT, HRT, and aeration intensity frequently emerge among the most influential predictors. However, their relative rankings vary across datasets, plant configurations, and modelling objectives. A recent full-scale study by Liang et al. applied CatBoost combined with XAI to an MBR treating food processing wastewater [49]. CatBoost achieved R^2^ = 0.8374. XAI analysis identified the food-to-microorganism (F/M) ratio and MLSS as the two most influential fouling predictors. This finding is consistent with mechanistic understanding. It also demonstrates that XAI-informed ML can provide actionable guidance for full-scale MBR operations, not only for controlled lab or pilot systems. To illustrate how SHAP outputs translate into operator actions, consider a hypothetical but physically realistic scenario at a municipal MBR plant. Note: the numerical values below are illustrative. They are not extracted from a specific published dataset. They are consistent with operational ranges and SHAP output structures reported in Liang et al. and Kovacs et al. A Random Forest model predicts a TMP exceedance within four hours. The SHAP explanation for this prediction identifies four feature contributions. MLSS = +2.1 kPa: mixed liquor concentration near its upper operating limit is the dominant risk driver. HRT = +1.4 kPa: short retention time is increasing organic loading to the membrane. Aeration intensity = −0.8 kPa: the current scouring rate is partially mitigating fouling risk. Feed flow rate = +0.6 kPa: elevated influent flow is compressing the cake layer. The SHAP values sum to the model output, confirming local accuracy. An operator reading this explanation identifies immediately that MLSS is the dominant risk factor. The current aeration rate is providing partial protection. The actionable response is to increase membrane scouring aeration and prepare for a relaxation cycle. This explanation uses the same process variables that operators monitor routinely. No specialist ML knowledge is required to act on it. This is the practical value of XAI in MBR operations: it tells the operator which specific condition is driving the risk and by how much [17,25,42]. This consistency is not merely a validation of known process understanding; it provides quantitative evidence that the ML models have learned physically meaningful representations of fouling dynamics rather than spurious correlations. In studies where SHAP global rankings diverge from mechanistic expectations. For example, when a SCADA timestamp feature is of high importance, it serves as an early warning of overfitting or data leakage, which can be detected and corrected before deployment [17,53].

Local SHAP values, computed for individual predictions, enable per-event explanation at the operational level. When a predictive model forecasts an imminent TMP exceedance, the associated SHAP values identify which specific process conditions are driving the prediction for that time step. An example being a combination of elevated MLSS (positive SHAP contribution), reduced HRT (positive), and adequate aeration (negative, stabilizing) with magnitudes that allow the operator to evaluate whether the prediction is consistent with their knowledge of the current operating state. Without interpretable local explanations, operators may be less willing to rely on model recommendations in safety or compliance-sensitive contexts [29].

Newhart et al. demonstrated the value of data-driven monitoring frameworks for MBR operational management, establishing that ML models trained on routine SCADA sensor streams can detect process upsets and deviations that precede measurable performance degradation [17]. Their work provided foundational evidence for integrating ML-based process monitoring into the MBR control infrastructure and identified the sensor variables most informative for state estimation. Viet and Jang applied AI-based predictive models to an OMBR system, demonstrating that draw solution concentration, feed water pH, and conductivity were the primary determinants of water flux and fouling resistance, with model predictions achieving R^2^ = 0.92–0.98 [22]. These feature-importance findings are consistent with the osmotic-driving-force principles governing OMBR performance, confirming that ML-derived variable rankings can recover a mechanistically correct process understanding even in complex coupled systems.

Progressive evolution of SHAP values across an MBR filtration cycle provides operational insights not accessible from static feature importance rankings. As fouling accumulates during a filtration cycle, SHAP attributions for flux setpoint and MLSS tend to increase monotonically, reflecting the growing cake resistance. In contrast, the SHAP contribution of membrane aeration may plateau or decrease as the cake layer transitions from a loose, shear-removable deposit to a compacted gel layer where scouring becomes less effective. Monitoring these temporal SHAP profiles in real time provides early warning indicators of the transition from manageable to critical fouling, enabling proactive intervention before TMP reaches cleaning thresholds [17,53].

### 4.3. LIME, Partial Dependence Plots, and Gradient-Based Methods

LIME (Local Interpretable Model-agnostic Explanations) generates locally linear surrogate models around individual predictions by perturbing input features and observing the effect on the model’s output [27]. For each prediction to be explained, LIME samples a neighborhood around the input point, weights samples by their proximity to the original input, and fits a simple interpretable model (typically linear regression or a shallow decision tree) to the sampled outputs. The coefficients of this local surrogate model explain the black-box model’s local behavior. LIME’s key advantage is computational efficiency: explanations can be generated in milliseconds, making it suitable for real-time operational dashboards where explanation latency is a constraint. Its limitation is inconsistency: explanations for similar inputs can vary significantly due to random sampling, and the choice of neighborhood radius affects explanation stability. The application of LIME specifically to MBR fouling and process control models is an emerging area of the literature with limited peer-reviewed examples, and a systematic comparison of LIME and SHAP performance in MBR contexts is a gap for future research.

Partial dependence plots (PDP) and individual conditional expectation (ICE) curves provide a global visualization of the marginal relationship between individual input features and model predictions [48]. PDPs show the expected model output as a function of a target feature, marginalized over the distribution of all other features; ICE curves disaggregate this by showing the relationship for each sample, revealing heterogeneity in the feature effect that is hidden in the averaged PDP. In MBR applications, PDP analysis of MLSS concentration typically reveals a non-linear threshold pattern, with fouling rates remaining relatively stable at MLSS below 10–12 g/L before increasing steeply, consistent with the empirically observed transition from dilute suspension dynamics to viscous, non-Newtonian sludge behavior above this concentration. ICE analysis further reveals that this threshold shifts with temperature and SRT, reflecting seasonal and operational variations in sludge filterability that complicate fixed-setpoint control [48].

Gradient-based attribution methods, originally developed for deep learning in computer vision and natural language processing, quantify the sensitivity of model outputs to input perturbations by analytically differentiating the computational graph [28,54]. Methods including vanilla gradients, integrated gradients (which average gradients along a path from a reference baseline to the actual input), and guided backpropagation have been applied to temporal neural network architectures, such as LSTMs and convolutional-recurrent hybrids, in water system monitoring applications. For MBR TMP prediction with LSTM models, gradient attribution maps identify which time steps carry the highest predictive information for the current TMP state. This typically corresponds to the 2–8 h immediately preceding the prediction window. This provides insight into the characteristic timescales of cake layer formation and consolidation, which are relevant for maintenance scheduling [28]. The application of these methods in MBR-specific deep learning models represents a frontier research area with significant practical potential. Table 3 summarizes the five principal XAI methods identified across the reviewed literature, comparing their explanation scope, model compatibility, computational cost, and MBR applications.

While XAI methods address the interpretability barrier to ML adoption, a parallel operational challenge in MBR systems concerns energy efficiency. Aeration for membrane scouring and biological treatment represents the dominant energy expenditure, and its optimization depends on the same complex, non-linear process relationships that ML models are well-suited to capture. The following section examines the evidence base for ML-driven energy optimization and establishes clear benchmarks against which future progress can be measured.

## 5. ML-Driven Energy Optimization in MBR Systems

### 5.1. Energy Consumption Structure and Optimization Targets

Energy cost is the primary operational expenditure for MBR systems beyond membrane replacement, and aeration is by far the dominant energy consumer. Verrecht et al. established, through mechanistic modelling benchmarked against two full-scale plants, that aeration for membrane scouring and biological treatment combined consumes 0.4–0.8 kWh/m^3^ [56]. Krzeminski et al. confirmed, through a broader empirical survey, that total specific energy consumption for MBR systems typically falls in the range 0.8–1.1 kWh/m^3^, with the distribution within this range primarily driven by aeration control strategy, membrane flux setpoint, and sludge settleability [15]. Germain et al. also demonstrated that specific energy demand varies substantially across MBR configurations and that benchmarking across plants requires careful normalization for influent strength and treatment objectives [57]. Conventional aeration control in MBR systems employs either fixed air flow rate setpoints (determined during commissioning trials) or simple proportional-integral (PI) feedback on dissolved oxygen concentration. Fixed setpoints are energy-inefficient because they maintain membrane scouring aeration at the maximum required rate regardless of actual fouling conditions, resulting in systematic over-aeration during clean membrane periods immediately after backwashing or chemical cleaning. DO-feedback control improves biological aeration efficiency but does not directly manage membrane-scouring aeration, leaving the dominant aeration energy component under open-loop control [36]. The fundamental challenge is that the optimal scouring aeration rate is not a fixed process parameter. It depends on the current fouling state (cake layer thickness and compressibility), mixed-liquor viscosity, flux setpoint, and membrane age. None of these variables are directly measurable in real time with standard SCADA instrumentation [58].

This gap creates the natural use case for ML-based aeration optimization: a model that predicts the minimum scouring aeration required to maintain TMP below the critical fouling threshold under the current process conditions, given a stream of SCADA sensor inputs. Such a model would enable dynamic aeration adjustment that reduces energy consumption during favorable conditions while automatically increasing aeration when fouling risk is elevated, precisely the control action that requires the kind of non-linear, multi-variable mapping that ML architectures excel at providing [17,38].

### 5.2. Confirmed Energy Reduction Evidence and Research Gap

A critical distinction must be made at the outset of this section. Energy savings reported in the MBR literature originate from two fundamentally different control approaches. The first is ASM-based mechanistic control, which uses process models such as ASM1 or ASM2d combined with PI feedback. The second is ML-based data-driven control, which uses trained algorithms to predict and minimise energy demand. These two approaches are not equivalent, and conflating them overstates the maturity of ML-driven energy optimisation. Before examining the available evidence, it is important to distinguish between these two categories of energy optimization outcomes reported in the MBR literature. The first comprises validated energy savings measured at full-scale operational plants using mechanistic or feedback control strategies. The second comprises projected savings reported in simulation studies or pilot-scale control experiments, where the specific control algorithm and boundary conditions vary across publications, and savings are not always directly attributable to ML. This distinction is critical for a fair assessment of what ML has demonstrably achieved in MBR energy management versus what remains to be validated at an operational scale.

The most directly comparable evidence of energy reduction in the current literature comes from Sun et al., who conducted activated sludge model (ASM) simulations combined with proportional-integral (PI) feedback control of aeration at a full-scale MBR installation [59]. This model-based control approach, which used ASM predictions of oxygen demand to dynamically adjust biological aeration setpoints, achieved a 20% reduction in aeration energy demand, lowering total specific energy consumption to 0.45 kWh/m^3^, compared with the 0.73 kWh/m^3^ pilot-scale baseline established by Verrecht et al. [14]. This result confirms that dynamic, model-informed aeration control can deliver meaningful energy savings at full scale. However, it is important to note that the control algorithm used by Sun et al. was ASM-based PI control rather than an ML approach per se. The 20% aeration reduction thus represents a validated benchmark for model-based control rather than a confirmed ML-specific outcome.

Dedicated ML-driven energy optimization studies specifically targeting MBR aeration demand remain limited in the current literature. The range of aeration savings sometimes referenced in broader MBR optimization literature (spanning from approximately 15% to more than 25%) derives primarily from simulation studies, design optimization analyses, and pilot-scale control experiments where the control algorithm varies across publications and the savings are not always attributable to ML specifically. The development, validation, and full-scale demonstration of ML-based aeration optimization for MBR systems using real-time SCADA data, closed-loop control, and rigorous energy accounting are identified as among the highest-priority research directions in this field. Table 4 consolidates the confirmed specific energy consumption benchmarks and optimization evidence from the primary studies identified in this review.

The 20% reduction in aeration energy reported by Sun et al. was achieved through an ASM-based PI feedback control strategy rather than a dedicated ML algorithm. This result constitutes a validated benchmark for model-informed control rather than a confirmed ML-specific outcome. No peer-reviewed full-scale study has yet demonstrated a quantified energy saving directly attributable to a deployed ML control algorithm in an MBR plant. The development, closed-loop validation, and full-scale demonstration of ML-based dynamic aeration control using real-time SCADA data and rigorous energy accounting, thus, remain the most immediately impactful open research priorities in the energy optimization domain.

Addressing both the fouling prediction and energy optimization challenges at operational scale requires a unified framework that integrates ML prediction, XAI transparency, and mechanistic process understanding into a continuously updated operational tool. Digital twin technology provides precisely this integration layer, and its application to MBR systems is examined in the following section.

## 6. Digital Twin Frameworks for MBR Systems

### 6.1. Architecture, Components, and Maturity Tiers

The digital twin concept was first formally articulated in manufacturing by Grieves [30] and has since been elaborated across diverse industrial domains, including aerospace, energy infrastructure, and smart manufacturing [32]. Fuller et al. identify three essential components shared across industrial DT implementations: a physical entity with instrumented sensing and actuation; a virtual entity that models the physical system across relevant timescales; and a data connection layer enabling continuous state synchronization between physical and virtual domains [31]. The virtual entity must be multi-fidelity. It combines high-accuracy physics-based models for slowly varying system states with faster data-driven models for dynamic operational variables. This balance maintains both computational feasibility and predictive accuracy [33].

For MBR systems, this DT framework has three layers. The first is the physical plant: its SCADA network, online sensors (DO, turbidity, pressure transducers, flow meters), and actuators (blowers, pumps, valves). The second is a hierarchical virtual model incorporating ASM mechanistic sub-models [34,35], resistance-in-series fouling models [36], ML-based dynamic TMP predictors trained on operational data [22,26], SHAP-based feature attribution modules [53], and energy balance sub-models [14,56]. The third is a real-time data pipeline, potentially cloud-hosted, that synchronizes sensor readings to the virtual model and feeds model outputs back to the control system [37]. Wang et al. review DT applications across the entire wastewater treatment lifecycle, identify MBR fouling management and aeration optimization as the highest-value integration targets, and demonstrate that DT approaches can generate plant-wide energy savings and effluent quality improvements through coordinated optimization of biological, filtration, and chemical dosing subprocesses.

In practice, MBR digital twin implementations can be stratified into three capability tiers, reflecting increasing integration depth and autonomous decision-making. Tier I (Descriptive) twins monitor and visualize real-time plant state through sensor dashboards, alarm management systems, and historical trend analysis, capabilities that are already commercially available and deployed at many full-scale MBR installations [5,15]. The rapid growth of DT research in the water sector supports this framing. A recent review of 147 studies spanning 2015 to May 2025 found that DT publications in the sector grew from one in 2015 to 41 in 2024 [60]. Of those 147 studies, 41 addressed wastewater treatment. This growth confirms that DT technology is moving from conceptual proposals toward structured implementation frameworks. Rodríguez-Alonso et al. demonstrated a practical microservices-based DT platform for a full wastewater treatment plant. It provides a concrete Tier I/II deployment reference using edge computing architecture [61]. Tier II (Predictive) twins forecast future plant states using trained ML models, including TMP trajectory, effluent quality, and energy demand 12–72 h ahead. This enables proactive rather than reactive operational decisions. This tier has been demonstrated in BSM-MBR simulation studies [24,36] and validated in isolation with full-scale data [26]. Tier III (Prescriptive) twins autonomously generate, evaluate, and implement operational decisions. These include aeration setpoint adjustments, flux control, and cleaning schedule optimization. Closed-loop simulation evaluates predicted consequences before any physical action is taken. No full-scale MBR implementation of Tier III with XAI integration has been documented in the literature, representing the primary frontier for the field [33,37].

### 6.2. XAI Integration in Digital Twin Decision Architecture

One of the most important architectural advances in higher-maturity MBR digital twins is the integration of explainable AI (XAI) modules as decision-support and transparency layers between the prediction engine and downstream human or control actions, improving interpretability, trust, and operational usability [62,63]. This explanation can be presented to the operator in interpretable visual formats such as a SHAP waterfall chart or a local ranked bar plot. It may also be translated into user-oriented summary text, enabling the operator to assess whether the prediction is plausible given the current process state before deciding whether to act on it [64].

First, within future DT-enabled MBR control architectures, the XAI layer could provide a near-real-time audit trail by logging the explanatory context associated with model-informed recommendations, thereby improving traceability, accountability, and post hoc review during internal audits, compliance assessments, or regulatory inspections [65]. Second, it enables informed operator override. Rather than simply accepting or rejecting an ML recommendation, operators can use the SHAP attribution to identify which specific variables are driving the prediction. They can then assess whether those variables reflect the true process state or a measurement artefact, enabling informed human oversight rather than blind rejection [17,66]. Third, shifts in explanation patterns, such as marked changes in SHAP ranking distributions, may also serve as interpretable warning signals of concept drift or operation outside the model’s familiar regime, thereby triggering human review before continued automated control [48,67].

The data and decision flow within a Tier II/III MBR digital twin with embedded XAI operates through five stages. In the first stage, raw sensor streams, including dissolved oxygen, transmembrane pressure, permeate flux, turbidity, temperature, and membrane aeration flow rate, are transmitted from the physical plant via SCADA at one-minute intervals to the DT data layer, where they are quality-checked, gap-filled by interpolation, and stored in a time-series database. In the second stage, a trained ML prediction engine, such as a random forest or LSTM model for TMP forecasting and a gradient boosting model for effluent quality estimation, generates predictions 12 to 72 h ahead from the current and recent sensor history. In the third stage, a SHAP-based explanation module computes per-feature attribution values for each prediction in real time, producing a ranked contribution plot that identifies which process variables are driving the current forecast and by how much. In the fourth stage, both the prediction and its SHAP explanation are presented on an interpretable operator dashboard, enabling informed human review before any control action is executed. In the fifth stage, accepted recommendations are passed to the control layer, which adjusts aeration setpoints, flux targets, or cleaning schedules as required, and the prediction, together with its explanation, is automatically logged to an audit trail for regulatory traceability and post-event review. This architecture ensures that XAI is structurally embedded in the decision pipeline rather than functioning as an optional post-hoc module.

Tao et al. [32] and Barricelli et al. [33] both identify interpretability as a fundamental design requirement for trustworthy industrial digital twins. They note that unexplained model recommendations under novel process conditions, even if quantitatively correct, will be systematically overridden by conservative operators. This negates the operational value of the twin. The incorporation of XAI into DT architecture is therefore not merely a regulatory convenience but a functional requirement for achieving the operator trust necessary for the transition from Tier II advisory to Tier III prescriptive operation. The convergence of ML prediction, XAI transparency, and mechanistic model consistency checking within a unified DT architecture represents the most complete available framework for intelligent, trustworthy MBR operation at the current state of the art [37]. Table 5 presents a structured overview of the three DT maturity tiers applicable to MBR systems, from descriptive monitoring through to closed-loop prescriptive control.

The three tiers can also be characterized by their evidence status. Tier I is commercially deployed at full municipal and industrial scale worldwide [5,15] and therefore represents validated operational technology. Tier II has been demonstrated in BSM-MBR simulation studies [36] and validated using full-scale SCADA datasets (Kovacs et al., 2022 [26]), but has not yet been deployed as a continuously operating closed-loop advisory system in a commissioned plant; its evidence status is therefore simulation-to-full-scale data validation. Tier III remains at the conceptual and architectural proposal stage: no full-scale MBR implementation combining Tier III prescriptive control with integrated XAI decision justification has been documented in the peer-reviewed literature as of December 2025. The transition from Tier II to Tier III represents the field’s primary frontier and is directly linked to the research gaps discussed in Section 7.

**Figure 3:**
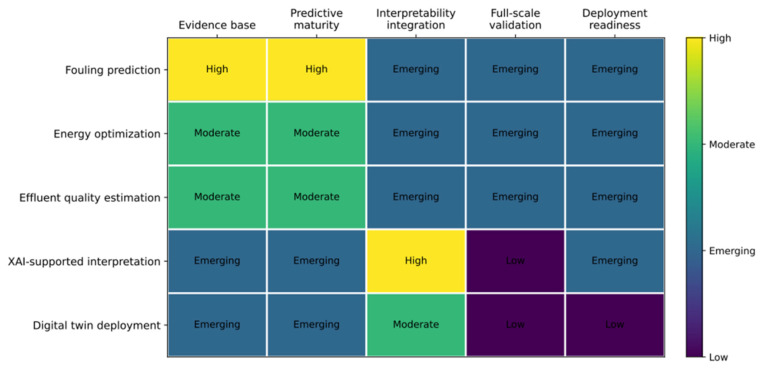
Research maturity heat map for intelligent wastewater membrane bioreactor (MBR) studies. The figure provides a qualitative synthesis of the reviewed literature across major application domains and deployment dimensions, indicating that fouling prediction is the most mature area.

To synthesize the current state of the field across application domains and deployment dimensions, Figure 3 presents a qualitative heat map of research maturity for ML, XAI, and digital twin applications in wastewater MBR systems.

In the heat map, each cell represents a qualitative maturity rating based on three criteria: the volume of peer-reviewed evidence available, the availability of full-scale operational validation, and the extent of documented deployment in commissioned water treatment facilities. High-rated cells indicate substantial evidence base supported by multiple independent studies with consistent results. Moderate-rated cells indicate an emerging evidence base that includes at least some pilot or full-scale validation. Emerging cells indicate that the application area is recognized, but evidence is confined to conceptual proposals, simulation studies, or a small number of laboratory-scale demonstrations. Low-rated cells indicate that no peer-reviewed evidence for that combination was identified in the structured search.

Three patterns are immediately apparent. First, fouling prediction shows the strongest evidence base and predictive maturity, both rated High, reflecting the substantial body of ML literature reviewed in Section 3. Nevertheless, its interpretability integration, full-scale validation, and deployment readiness are all rated Emerging, indicating that predictive capability has substantially outpaced practical implementation. Second, XAI-supported interpretation shows a high-rating for-interpretability integration, as expected for a technology whose primary function is interpretability, but Low full-scale validation and Emerging deployment readiness, confirming that XAI application in MBR systems remains largely an academic exercise. Third, digital twin deployment shows the lowest overall maturity profile, with Emerging or Low ratings across all five dimensions, reflecting the absence of full-scale operational deployments documented in the peer-reviewed literature. Each low-maturity cell in the figure maps directly to one or more of the nine research gaps identified in Section 7, providing a visual roadmap for prioritizing future research investment.

## 7. Research Gaps and Future Directions

This review reveals nine interconnected research gaps whose resolution is necessary before XAI-DT integration can achieve operational deployment at scale in MBR facilities. The first five gaps were identified in the original review. Four additional gaps were identified in response to reviewer comments and are presented as Gaps 6 through 9.

The first and most fundamental gap is the scarcity of benchmark datasets. The vast majority of reviewed studies trained and validated models on single-facility datasets, typically collected over weeks to months, precluding evaluation of cross-site generalization. When models trained on one MBR installation are applied to a second facility with a different membrane module configuration, influent composition, or operating regime, performance typically degrades significantly, a consequence of a dataset shift documented in the broader ML environmental engineering literature. The wastewater ML community currently lacks the equivalent of the standardized benchmark datasets that enabled rapid, systematic progress in computer vision, natural language processing, and genomics over the past decade. The development of openly accessible, multi-facility MBR operational datasets encompassing diverse scales, configurations, wastewater types, and climate zones is a key requirement for rigorously evaluating and improving ML model generalizability [17,24,47]. The BSM-MBR platform [36,50] provides a valuable simulation-based benchmark. However, simulation data remain valuable for benchmarking, but they do not fully reproduce the non-stationary, noisy, and operationally constrained conditions of real plant data.

Beyond dataset size, the capacity for cross-site generalization poses an equally fundamental challenge. When a model trained at one MBR installation is applied to a second facility operating under different conditions, predictive performance typically degrades because the statistical relationship between input features and fouling outcomes differs between plants in ways that single-site training data cannot capture. Dataset shift has several primary sources. These include differences in membrane module geometry between hollow-fiber and flat-sheet configurations, sludge microbiology shaped by local wastewater composition and temperature, and the specific SRT and HRT operating windows maintained at each facility. Industrial trade waste discharge events, which introduce rapid and large disturbances not captured in routine training data, are another key source. Transfer learning, in which a model pre-trained on a data-rich source facility is fine-tuned using a small amount of data from a target plant, and domain adaptation, which explicitly minimizes the distributional discrepancy between the source and target feature spaces, offer practical methodological pathways toward generalizable MBR ML models. Both approaches have demonstrated success in related environmental engineering and water quality monitoring applications [17,47]. They should be prioritized in future multi-facility MBR studies, ideally using openly accessible benchmark datasets as the shared testbed.

The second gap is the near-universal absence of uncertainty quantification in MBR ML predictions. Very few reviewed studies reported prediction intervals or confidence bounds alongside point estimates. This is a critical gap, since uncertainty is directly actionable in operational contexts. A control system with a TMP forecast uncertainty of ±2 kPa will respond differently than one facing ±12 kPa uncertainty. Yet the current literature overwhelmingly provides only deterministic point predictions. Bayesian neural networks, which maintain probability distributions over network weights rather than point estimates, provide principled uncertainty quantification but are computationally demanding; conformal prediction methods, which provide distribution-free coverage guarantees under exchangeability assumptions, offer a computationally efficient alternative increasingly used in safety-critical engineering applications [28,48]. Neither approach has been systematically evaluated in the MBR ML context. For Tier III prescriptive DT operation, where control decisions are executed, automatically calibrated uncertainty bounds are not merely desirable but operationally essential for safe autonomous control.

The third gap is the lack of full-scale DT deployments with integrated XAI. Of the DT-focused publications identified in this review, all described simulation-based or laboratory-scale frameworks; none documented a complete Tier II or Tier III DT implementation at full municipal scale with contemporaneous XAI explanation generation and operator integration. The infrastructure requirements for full-scale deployment are substantial and multidimensional. Technical requirements include sensor calibration, data pipeline reliability, computational infrastructure, and cybersecurity. Organizational requirements include operator training, change management, and contractual liability frameworks for automated control. These organizational factors have not been well documented in the academic literature [33,37]. Structured collaboration between water utilities, technology providers, and academic research groups of the kind that has produced the full-scale ML validation work of Kovacs et al. [26] is needed to generate the deployment evidence base that will underpin regulatory and commercial adoption.

The fourth gap concerns dynamic influent characterization. Standard SCADA instrumentation measures bulk wastewater quality parameters (COD, BOD, TSS, turbidity, DO, pH, and conductivity) that provide only an aggregate characterization of the mixed liquor entering the biological system. Industrial trade waste discharge events, stormwater infiltration during rainfall, and diurnal variation in domestic sewage composition all introduce rapid, large-magnitude changes in influent characteristics that are poorly represented by daily composite samples or slow-response online sensors [3,22]. These uncharacterized disturbances are a primary source of MBR fouling events that are difficult to predict from standard sensor arrays. Integration of high-frequency online spectroscopic sensors (UV-Vis, fluorescence spectrophotometry), emerging optical coherence tomography for real-time cake layer characterization, and even molecular tools for microbial community fingerprinting into the DT sensor layer would substantially improve dynamic influent characterization and ML model predictive coverage of these high-consequence disturbance events.

The fifth gap concerns the regulatory dimension of XAI adoption. The theoretical case for XAI as a tool for building regulatory trust in ML-based water system management is well-developed in the academic literature [29,51]. However, no published study has empirically documented a case where SHAP explanations or equivalent XAI outputs directly influenced a permit condition, regulatory inspection outcome, operational license amendment, or other formal regulatory action in the water sector. This represents a critical missing link between the technical capability demonstrated in academic research and the regulatory permission structures that govern operational water systems. Proactive engagement between the water engineering community and environmental regulators to develop agreed-upon evidence standards for XAI validation, define acceptable use cases for automated ML-based control, and establish audit-trail requirements is a prerequisite for the transition from research demonstration to licensed operational deployment that the field currently needs. A sixth gap concerns the economic cost of ML model development and retraining. Developing a production-grade ML model for an MBR plant requires data infrastructure, engineering expertise, and computational resources. Retraining costs recur whenever influent characteristics shift, membrane modules are replaced, or plant configuration changes. These costs are rarely reported in academic studies. Future work should document the full cost of model development, validation, and maintenance cycles. This information is essential for water utilities evaluating whether ML-based control is economically justified relative to simpler alternatives. To contextualise this gap, a realistic deployment cost structure for an ML-based fouling prediction system at a medium-sized MBR plant includes the following components. Initial development covers sensor audit and data infrastructure (typically several months of SCADA historian cleanup), model development and cross-validation, and regulatory documentation for process change approval. Ongoing costs include periodic model retraining, edge computing hardware, IT security, and operator training. No peer-reviewed study has yet reported a full cost–benefit analysis for a deployed ML system in an MBR plant. This absence of data is itself a finding. Future studies should report total development cost, validation cost, and annual maintenance cost alongside model performance metrics. Without this information, water utilities cannot assess whether ML-based control is economically justified relative to simpler alternatives such as PID controllers. Until such data are available, practitioners should commission site-specific feasibility assessments before committing to ML-based control deployment. A seventh gap concerns the human capital requirements for operating a DT and XAI system. A functioning Tier II or Tier III digital twin requires staff with expertise in data engineering, ML operations, and process control. Most water utilities lack this skill set in-house. The gap between research-grade demonstrations and practical utility deployment is partly a workforce gap, not only a technology gap. Studies should explicitly report the staffing and training requirements of proposed DT frameworks. An eighth gap concerns the limited comparison of ML-based control with established non-ML alternatives. Proportional–integral–derivative (PID) controllers and fuzzy logic controllers are well-understood, low-cost, and already deployed in most MBR facilities. Very few reviewed studies benchmarked ML models against these baselines under equivalent operating conditions. Without such comparisons, the incremental benefit of ML over conventional control cannot be quantified. Future studies should include PID and fuzzy logic as mandatory baselines when claiming performance improvements. A ninth gap concerns the carbon and energy footprint of ML model computation. Training deep learning models such as LSTM networks or Transformers on large SCADA datasets consumes substantial electrical energy. Inference on edge hardware adds ongoing energy demand. For MBR systems deployed specifically to reduce environmental impact, this computational footprint is a legitimate concern. Future studies should report training energy consumption and inference energy per prediction alongside model performance metrics.

## 8. Conclusions

This comprehensive review has synthesized evidence from peer-reviewed sources on machine learning, explainable artificial intelligence, and digital twin applications in membrane bioreactor wastewater treatment. The following principal conclusions address the four review objectives stated in Section 1:

- ML models for MBR fouling and TMP prediction have matured substantially over the review period. Ensemble methods (particularly RF) and kernel-based approaches (LSSVM) achieve R^2^ = 0.85–0.99 across laboratory, pilot, and full-scale systems, consistently outperforming standard ANN architectures. Full-scale RF validation on 80,000+ operational samples (R^2^ = 0.927–0.996, RMSE = 0.264–0.904 kPa) represents a strong historical validation result. It is important to clarify, however, that historical validation on archived SCADA data is not equivalent to operational deployment validation. Closed-loop performance under live conditions, including sensor drift, noise, and data latency, has not yet been demonstrated. Site-specific validation and governance safeguards remain prerequisites before deployment in operational MBR monitoring systems.
- SHAP-based analyses in MBR studies frequently identify MLSS, SRT, HRT, and aeration intensity as dominant predictors. These findings simultaneously validate mechanistic understanding and provide operator-actionable evidence of the variables most critical for fouling control [17,25,42]. SHAP’s dominance across XAI-inclusive publications reflects both its theoretical grounding in cooperative game theory and its practical usability for engineering interpretation.
- Model-based and feedback-control approaches have confirmed up to 20% reductions in aeration energy demand at full-scale MBR installations [59], establishing a validated benchmark for model-informed control. Dedicated ML-specific energy optimization demonstrations remain limited and represent the most immediately impactful research gap for operational energy efficiency.
- Digital twin frameworks integrating mechanistic ASM sub-models with ML corrections provide the architecture for Tier II predictive and Tier III prescriptive MBR management. The embedding of XAI decision-transparency modules within the DT architecture is a functional requirement, not merely a desirable feature for achieving the operator trust necessary for autonomous prescriptive control. No full-scale MBR Tier III deployment with integrated XAI has yet been documented, which represents the field’s primary frontier.
- Nine critical research gaps require systematic investigation: standardized multi-facility benchmark datasets; calibrated uncertainty quantification in operational ML predictions; full-scale DT deployment evidence; dynamic integration of influent characterization technology; empirical evaluation of XAI within regulatory acceptance frameworks; transparent reporting of ML model development and retraining costs; workforce capacity building for DT and XAI operations at water utilities; systematic benchmarking of ML-based control against PID and fuzzy logic baselines; and reporting of computational energy footprints for deep learning models deployed in environmental systems.

The convergence of interpretable ML prediction, XAI transparency, and real-time digital twin simulation represents the most promising near-term pathway for intelligent, trustworthy MBR operation. Realizing this vision will require sustained, coordinated investment in open data infrastructure, uncertainty-aware model pipelines, full-scale deployment partnerships, and engagement with regulatory frameworks, contributions that must come from the water engineering, data science, and water governance communities acting in concert.

## Figures and Tables

**Table 1:** Confirmed ML model performance for MBR fouling and TMP prediction.

ML Algorithm	Target Variable	Scale	Best R^2^	Approx. Dataset Size	External Validation	RMSE/MSE	Reference
ANN (MLP + RBF)	TMP/permeability	Pilot	Satisfactory	N/R; 60-day campaign	None (train–test split)	N/R	[23]
ANN (backprop)	TMP, AO-MBR	Pilot	0.850	N/R; pilot-scale	None (train–test split)	N/R	[25]
LSSVM (best)	Fouling resistance	Lab	0.990	N/R; lab-scale	None (train–test split)	MSE = 0.0002	[42]
ANN-MLP	Fouling resistance	Lab	Lower than LSSVM	N/R; lab-scale	None (train–test split)	>LSSVM	[42]
AI models (OMBR)	Water flux + fouling	Lab	0.92–0.98	N/R; lab OMBR	None (train–test split)	Reported	[22]
RF (best)	TMP, full-scale WWTP	Full-scale	0.927–0.996	>80,000 samples	None (single plant)	0.264–0.904 kPa	[26]
LSTM	TMP, full-scale WWTP	Full-scale	Lower than RF (no exact value reported)	>80,000 samples	None (single plant)	Higher than RF	[26]
ANN	TMP, full-scale WWTP	Full-scale	Lower than RF (no exact value reported)	>80,000 samples	None (single plant)	Higher than RF	[26]

**Table 2:** Critical comparison of principal ML algorithms applied to MBR fouling and TMP prediction.

Algorithm	Category	Key Strength	Key Limitation	Best R^2^	Bias Risk ^1^	Reference
ANN-MLP + RBF	Shallow ANN	Fast convergence; handles non-linear input-output relationships	No quantitative R^2^ reported; generalizability unvalidated	Not reported	HIGH	[23]
ANN (backprop)	Shallow ANN	Established architecture; practical for pilot-scale use	R^2^ = 0.850 only; moderate accuracy; no uncertainty quantification	0.850	HIGH	[24]
LSSVM	Kernel-based	Highest R^2^ in lab setting (0.99); robust on small data; built-in sensitivity analysis	Does not scale to large datasets; no temporal modeling capability	0.990	HIGH ^2^	[42]
AI models (OMBR)	Various	Captures osmotic driving force dynamics; R^2^ = 0.92–0.98	Small lab dataset; single facility only; no external validation	0.92–0.98	HIGH	[22]
Random Forest	Ensemble	Best accuracy at full scale; robust to outliers; built-in feature importance; handles mixed data types	Memory-intensive; global feature importance only; single-plant validation	0.927–0.996	MODERATE ^3^	[26]
LSTM	Deep learning (RNN)	Captures long-range temporal fouling dependencies; suited to time-series TMP	Requires large datasets; high compute demand; less accurate than RF in same study	Lower than RF	MODERATE ^3^	[26]
CatBoost + XAI	Gradient boosting	Strong full-scale performance; XAI identifies dominant fouling drivers (F/M, MLSS)	Moderate R^2^ (0.8374) on noisy industrial data; single food-processing plant	0.8374	MODERATE ^3^	[49]
MBR-Net (custom DL)	Deep learning	Real-time IoT-integrated prediction; R^2^ > 0.87 on two independent test sets; one-day-ahead forecasting	Limited by data availability; single facility type validated	>0.87	LOW ^4^	[50]

^1^ Bias risk rated per Reviewer 2 criteria: High = no external validation + dataset < 500 samples; Moderate = large dataset but single-facility validation only; Low = validated on ≥2 independent test sets. ^2^ LSSVM trained on lab-scale data only; dataset size not reported but consistent with <500 samples given lab campaign. ^3^ Single municipal or industrial plant; no cross-site validation performed. ^4^ MBR-Net validated on two independent test sets from the same full-scale facility; cross-site generalization not yet demonstrated.

**Table 3:** XAI methods applied in MBR and related water treatment studies: characteristics and applications.

XAI Method	Explanation Scope	Model Compatibility	Comp. Cost	Applications in MBR/Water Treatment	References
SHAP	Local + Global	Model-agnostic	Medium–High	Fouling prediction, energy optimization, effluent quality	[17,53]
LIME	Local	Model-agnostic	Low–Medium	Anomaly detection, water quality classification	[27,55]
PDP/ICE	Global	Model-agnostic	Low	Feature threshold identification, operating curve analysis	[48]
Integrated Gradients	Local (temporal)	Neural networks	Low	LSTM fouling forecasting, temporal attribution	[28,54]
ANCHORS	Local (rule-based)	Model-agnostic	High	Conceptual: operational rule extraction	[27]

**Table 4:** Confirmed energy benchmarks and optimization evidence for MBR systems.

Scale	Method	Key Finding	Confirmed Energy Metric	Reference
Full-scale	Mechanistic energy model	Model validated within 20% of all plant parameters	Aeration: 0.4–0.8 kWh/m^3^	[56]
BSM-MBR simulation	ASM scenario optimization	Energy reduced by SRT/recirculation tuning	Pilot baseline: 0.73 kWh/m^3^	[14]
Multiple full-scale	Empirical survey	Benchmarking across diverse MBR plants	Typical range: 0.8–1.1 kWh/m^3^	[15]
Full-scale	ASM + PI feedback control	Dynamic aeration control reduced blower demand 20%	Total: 0.45 kWh/m^3^ (−20% aeration)	[59]

**Table 5:** Digital twin maturity tiers for MBR systems with capability and implementation characteristics.

Tier	Capabilities	Data Requirements	Implementation Complexity	Status in MBR Literature	References
I—Descriptive	Real-time monitoring, dashboards, alarm management	SCADA, online sensors	Low	Commercially deployed	[5,15]
II—Predictive	TMP forecast, effluent quality prediction, fault detection, 12–72 h ahead	SCADA + lab analytics + trained ML	Medium	Validated in simulation and full-scale data	[24,26,36]
III—Prescriptive	Closed-loop autonomous optimization, what-if scenario testing, XAI decision justification	Full DT + actuators + XAI + safety validation	High	No full-scale MBR deployment documented	[33,37]

## Data Availability

No new data were created or analyzed in this study.

## Supplementary Materials

The following supporting information can be downloaded at https://www.mdpi.com/article/10.3390/membranes16050181/s1. Table S1: Study-level data extraction for reviewed ML, XAI, and DT studies on MBR systems.
