# Supplementary material for: Wastewater Membrane Bioreactors: A Comprehensive Review of Explainable Artificial Intelligence and Digital Twin Applications
Source: Membranes (Basel). 2026 May 21;16(5):181. doi: 10.3390/membranes16050181 (PMC13208125; doi:10.3390/membranes16050181)
Supplement: Supplementary file 1 [file membranes-16-00181-s001.zip › membranes-4280886-supplementary.pdf]

Supplementary Table S1. Study-level data extraction for reviewed ML, XAI, and DT studies on MBR systems.

Scale	Wastewater Type	ML/Modelling Algorithm	Target Variable	Unit	Reported Value Range	Performance Metric(s)	Approx. Dataset Size	External Validation	Ref
Pilot (submerged MBR)	Municipal	ANN-Genetic Algorithm (ANN-GA)	TMP; Membrane fouling	kPa; N/R	Not reported	Qualitative satisfactory performance (exact metrics N/R)	N/R; pilot-scale campaign	None (train-test split)	[23]
Pilot	Domestic municipal	ANN (backprop)	TMP	kPa	Not reported	$R^2 = 0.850$	N/R; pilot-scale campaign	None (train-test split)	[25]
Laboratory	Synthetic / lab	LSSVM	Fouling resistance	m^{-1}	Not reported	$R^2 = 0.990$; MSE = 0.0002; MAAPE = 3.18	N/R; lab-scale; est. <500 samples	None (train-test split)	[42]
Laboratory	Synthetic / lab	ANN-MLP	Fouling resistance	m^{-1}	Not reported	$R^2 < 0.990$; MSE > 0.0002 (exact N/R)	N/R; lab-scale; est. <500 samples	None (train-test split)	[42]
Laboratory	Synthetic / lab	ANN-PSO	Fouling resistance	m^{-1}	Not reported	$R^2 < 0.990$ (exact N/R)	N/R; lab-scale	None (train-test split)	[42]
Laboratory / pilot	Mixed industrial + municipal (UAE)	ANN	COD; BOD; Turbidity	mg/L; mg/L; NTU	Not reported	Performance reported qualitatively; exact metrics N/R	N/R	None (train-test split)	[43]
Laboratory	Municipal (OMBR)	Multiple AI models	Water flux; Fouling resistance	L/m²/h; m^{-1}	Not reported	$R^2 = 0.92–0.98$ (best model)	N/R; lab OMBR campaign	None (train-test split)	[22]
Full-scale municipal WWTP	Municipal	Random Forest (RF)	TMP (BBP, DBP, ABP stages)	kPa	Not reported	$R^2 = 0.927–0.996$; RMSE = 0.264–0.904 kPa	>80,000 samples; 4 years	None (single plant only)	[26]
Full-scale municipal WWTP	Municipal	LSTM	TMP (BBP, DBP, ABP stages)	kPa	Not reported	R^2 < RF; RMSE > RF (exact N/R)	>80,000 samples; 4 years	None (single plant only)	[26]
Full-scale municipal WWTP	Municipal	ANN	TMP (BBP, DBP, ABP stages)	kPa	Not reported	R^2 < RF; RMSE > RF (exact N/R)	>80,000 samples; 4 years	None (single plant only)	[26]
Full-scale (review scope)	Municipal WWTP (review scope)	Review of multiple ML and data-driven methods	Process performance; upset detection (review scope)	Various	N/A (review)	Review paper: no primary performance metric reported by authors	N/A (review paper)	N/A (review paper)	[17]
Laboratory / pilot	High-salinity industrial	SHAP-CatBoost	Effluent NH_4^+-N; Total Nitrogen (TN)	mg/L	Not reported	$R^2 = 0.88$ (NH_4^+-N); $R^2 = 0.91$ (TN); RMSE = 4.27 (NH_4^+-N); RMSE = 4.35 (TN)	N/R	None (train-test split)	[57]
Full-scale WWTP	Municipal	MBR-Net (custom deep learning)	Membrane permeability (irreversible fouling)	LMH bar^{-1}	Not reported	$R^2 > 0.87$; MAPE < 6.45%; MAE < 3.71 LMH bar^{-1} (two independent test sets)	Full-scale operational data; size N/R	Two independent test sets (same facility)	[62]
Laboratory / pilot	Domestic municipal	Decision tree regression (best); SVR; LR compared	TMP	kPa	Not reported	$R^2 = 0.99$ (decision tree; best)	N/R; est. <500 samples	None (train-test split)	[63]
Full-scale	Food processing industrial	CatBoost + XAI	Specific flux (flux/TMP)	L/m²/h/kPa	Not reported	$R^2 = 0.8374$ (best model)	Full-scale operational data; size N/R	None (single plant only)	[67]
Full-scale (two plants)	Municipal	Mechanistic energy model	Aeration energy consumption	kWh/m³	0.4–0.8 kWh/m³ (aeration)	Model validated within 20% of plant measurements	Two full-scale plants	Validated against plant data	[52]
Full-scale (simulated dynamic profile)	Municipal (simulated)	ASM1-based cost and energy sensitivity analysis	Total energy demand (opex)	kWh/m³	Not reported as single value; scenario-dependent	Cost sensitivity analysis (NPV); energy as component of opex	Simulated dynamic influent profile; ASM1	N/A (simulation/modelling)	[14]

Multiple full-scale (review scope)	Municipal (diverse)	Review / benchmarking survey of full-scale MBR energy data	Total specific energy consumption	kWh/m³	Reported range: 0.4–3.0 kWh/m³ for full-scale municipal MBRs (immersed membranes: 0.4–1.0 kWh/m³)	Benchmarking review; not a single primary experiment	Multiple full-scale plants surveyed in review	Cross-plant comparison (review)	[15]
Full-scale	Municipal	ASM + PI feedback control	Aeration energy; Total energy	kWh/m³	Total: 0.45 kWh/m³ post-control (vs 0.73 baseline)	20% reduction in aeration energy demand	Full-scale operational data	Validated at full-scale plant	[55]

N/R = Not Reported. CI = Confidence Interval. Studies are ordered by primary application domain (fouling/TMP prediction, effluent quality, energy optimisation).
